# Chronic appendicitis; the overlooked cause of chronic abdominal pain: Case report

**DOI:** 10.1016/j.ijscr.2024.110593

**Published:** 2024-11-12

**Authors:** Eskinder Amare Assefa, Yonas Girma Shumiye, Abel Shiferaw Tesfaye, Anatia Kifle Alemu, Zekarias Seifu Ayalew

**Affiliations:** a Yehuleshet Medical and Surgical Center, Department of Surgery, PO BOX: 13682, Addis Ababa, Ethiopia; b Addis Ababa University, College of Health Sciences, School of Medicine Department of Pathology; c Addis Ababa University, College of Health Sciences, School of Medicine Department of Surgery; d Yekatit 12 Hospital Medical College

**Keywords:** Case report: chronic abdominal pain, Chronic appendicitis, Appendectomy

## Abstract

**Introduction:**

Acute appendicitis is a surgical emergency and the diagnosis is straightforward in majority of the cases. However, chronic appendicitis, a relatively rare cause of chronic abdominal pain, presents a unique challenge due to its atypical presentation. This rarity underscores the need for vigilance and thoroughness in our practice to avoid misdiagnosis or delay in diagnosis.

**Case presentation:**

We present a case of chronic appendicitis in which a 30-year-old male patient presented with a nine-month history of chronic abdominal pain with mild right lower quadrant tenderness. Abdominal ultrasound suggested a feature of appendicitis, and the patient underwent appendectomy; finally, the diagnosis was confirmed on the histopathologic exam.

**Discussion:**

Chronic appendicitis, a relatively rare condition, is often diagnosed late due to a lack of well-developed diagnostic criteria and atypical presentation. However, using abdominal U/S and CT scan imaging plays a crucial role in assessing chronic appendicitis, highlighting the importance of these tools in our practice. In many cases, the ultimate diagnosis is confirmed after a histopathology exam, reinforcing the need for a comprehensive approach to diagnosis.

**Conclusion:**

Chronic appendicitis can have an atypical presentation with milder symptoms, and it should be considered in the differential diagnosis of patients presenting with chronic right lower quadrant abdominal pain. Abdominal U/S is an important imaging modality in assessing suspect cases of chronic appendicitis.

## Highlights

• Chronic appendicitis is relatively rare pathology, accounting for 1.5% of all cases of acute appendicitis.
• Patient usually present with chronic or recurring right lower quadrant abdominal pain accompanied by tenderness.
• The diagnosis is often difficult due to lack of well-developed diagnostic criteria, the atypical presentations and physicians’ unfamiliarity with the case.
• Abdominal U/S is important for initial assessment, and abdominal CT is the imaging modality of choice for chronic appendicitis.
• In several cases, the diagnosis of chronic appendicitis is confirmed with histopathologic examination and appendectomy is the treatment of choice, which can result in resolution of the symptoms in majority of the cases.

## Abbreviations

CRP: C-Reactive Protein
CT: Computed Tomography
ESR: Erythrocyte Sedimentation Rate
SCARE: Surgical CAse REport
U/S: Ultrasound
WBC: White Blood Cell

## 1 Introduction

Acute appendicitis is a relatively common surgical pathology with lifetime cumulative risk ranging from 7 to 9 % [1,2]. In classical cases, its diagnosis is direct, presenting with initial peri-umbilical pain that shifts to the right lower quadrant of the abdomen associated with anorexia, nausea, and tenderness on the right lower quadrant of the abdomen [3]. On the contrary, the diagnosis of chronic appendicitis is difficult and delayed due to the atypical presentation and clinicians' inexperience towards the condition [4,5]. It is relatively rare, with an estimated incidence of 1.5 % of all cases of appendicitis [6]. Clinically chronic and acute appendicitis can have similar presentation and imaging finding. However, patient with chronic appendicitis will have longer duration and less intense abdominal pain than patient with acute appendicitis [1,3]. The diagnosis is usually difficult because of milder symptoms, a lack of well-developed diagnostic criteria, and doubt of clinicians about its existence [4,5,7]. Clinically, chronic or recurrent abdominal pain of more than seven days is used to differentiate chronic appendicitis from acute one.

However, in most cases, the ultimate diagnosis of chronic appendicitis is made based on histopathologic examination results [8]. In the vast majority of patients with clinically and pathologically confirmed chronic appendicitis (81.8 %–93.8 %), Appendectomy results in the resolution of their symptoms [9,10]. Herein, we present the case of a 30-year-old male patient with chronic appendicitis who presented with a complaint of chronic abdominal pain and emphasized the role of abdominal ultrasound in assessing the condition.

This case report is reported according to SCARE guidelines [11].

## 2 Case presentation

A 30-year-old male patient presented to our medical and surgical center with a complaint of right lower quadrant abdominal pain of 9 months duration. The pain is a severe dull aching type associated with the intermittent piercing type of peri-umbilical pain and less frequently with vomiting of ingested matter. The patient experiences the attack approximately once or twice a week, lasting for a day, and it resolves spontaneously with no intervention. There is no aggravating or relieving factor. He has no fever, urinary complaint, bowel habit change, or weight loss. For this complaint, the patient has repeated visits to different health facilities but was told to have non-specific abdominal pain with no further intervention.

**Fig. 1:**
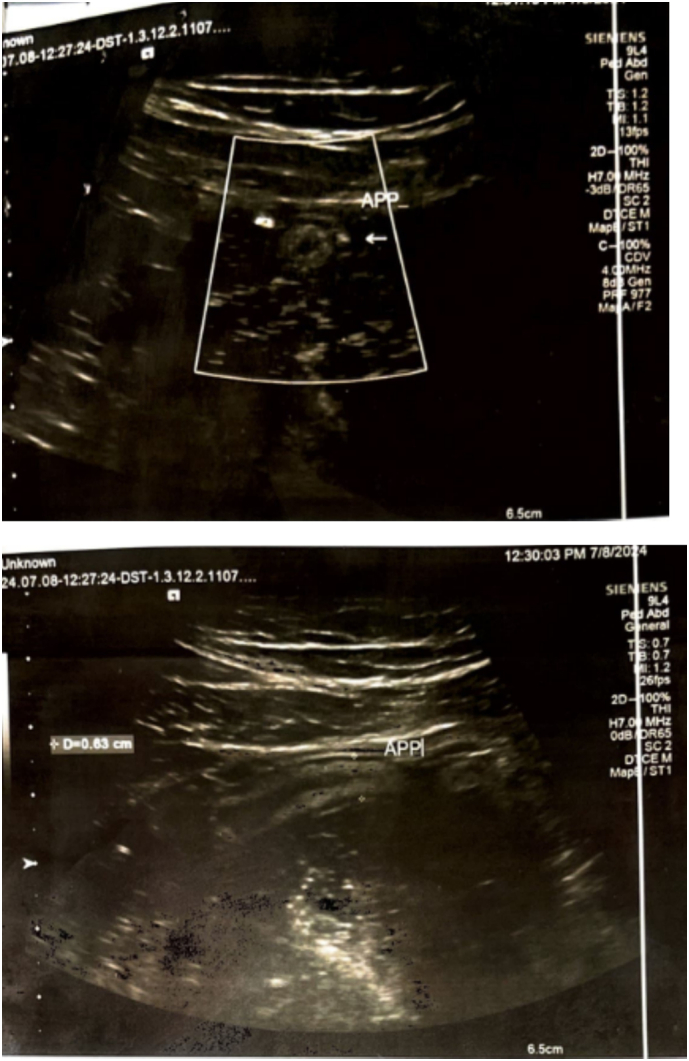
Abdominal ultrasound shows appendiceal wall thickening and increased wall to wall diameter.

On examination, the patient had stable vital signs and direct tenderness over the right lower quadrant of the abdomen but no rebound tenderness. Lab tests were done, and he has a WBC count of 6.6 × 10^3^/L, hemoglobin was 14 g/dl, and platelet count was 284 × 10^3^/L. CRP and ESR were within normal range, and stool exam and urine analysis were nonrevealing. Initial abdominal U/S showed an echogenic wall of the appendix that was partially compressible and measured 7 mm in diameter with slight probe tenderness. The radiologist suggested correlating the imaging findings with clinical findings. The patient was closely followed for two days, and a second U/S was done with a different hand, which revealed diffuse appendiceal wall thickening with prominent and hyperechogenic submucosa. The appendix was partially compressible with an outer wall-to-wall diameter of 6.3 mm, and there was no free fluid, hence suggesting chronic appendicitis (Fig. 1).

Considering the atypical presentation of the patient and the relative rarity of chronic appendicitis, an abdominal CT scan was ordered. Still, the patient declined due to the financial burden of repeated imaging and consented to undergo surgery based on the available imaging results.

**Fig. 2:**
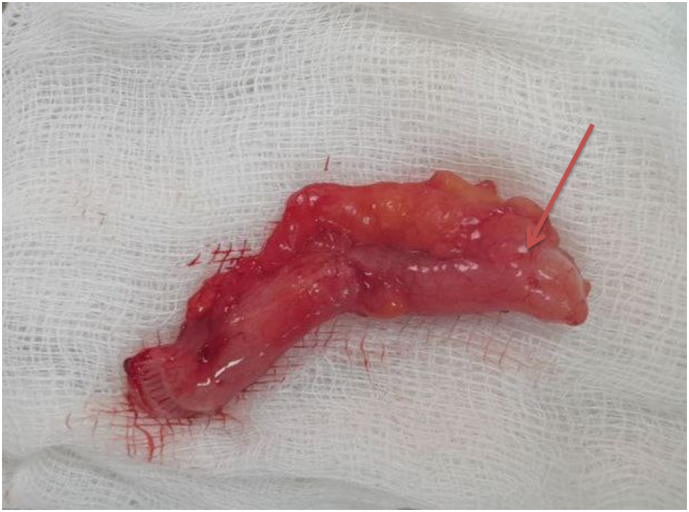
Gross appearance of the appendix after appendectomy showing scarred appendix with thickened tip (red arrow).

**Fig. 3:**
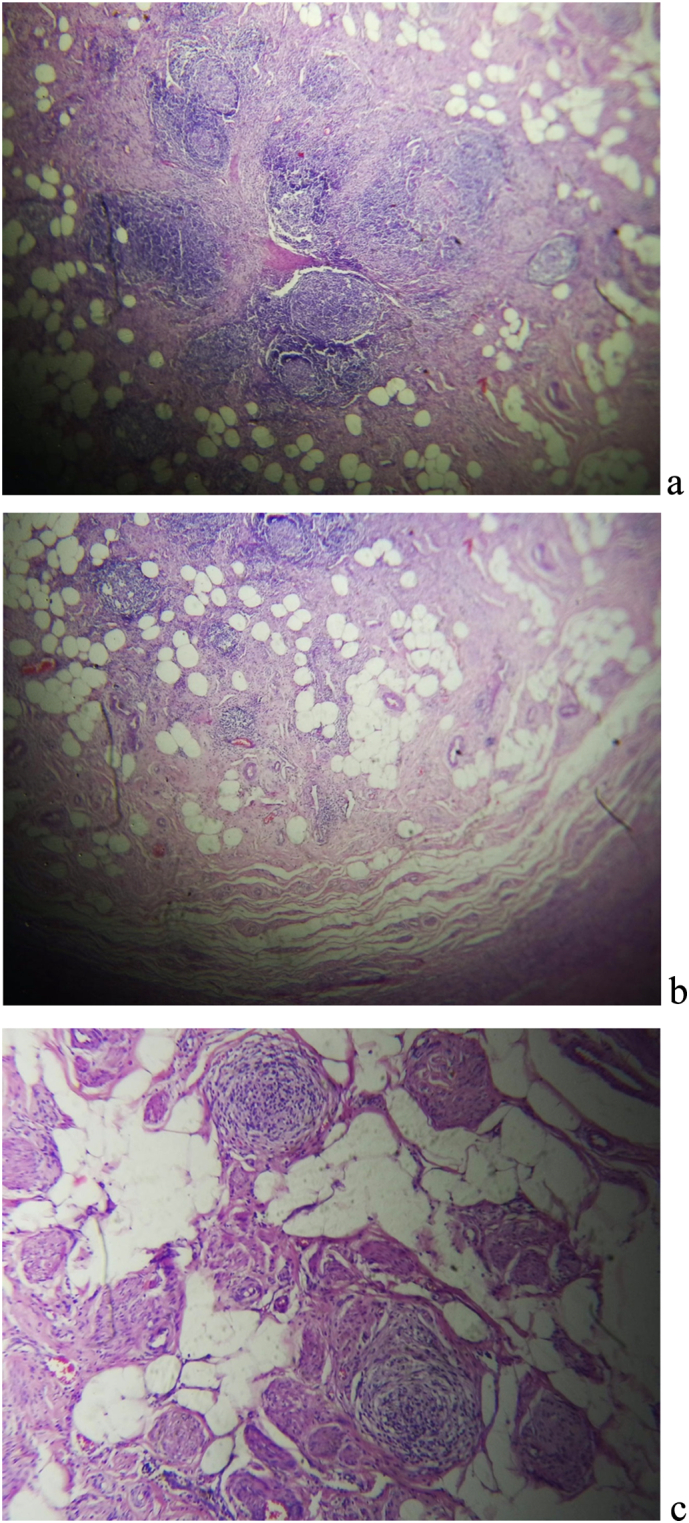
Histopathologic image of the removed appendix, a) Low power view of the appendix showing trans mural inflammatory infiltrates with trapped mesenteric fat b) Low power view showing mural fibrosis with inflammatory infiltrates forming lymphoid follicles. C) High power view displaying lymphoid follicles with fibrosis and entrapped mesentery.

An appendectomy was done, and the appendix grossly appeared hyperemic, dilated, and scarred with a thickened tip (Fig. 2). The patient had a smooth course after the surgery and was discharged on the second postoperative day. Then, the appendix was subjected to histopathologic examination, and the diagnosis of chronic appendicitis was confirmed (Fig. 3). On subsequent outpatient follow-ups at 1st week, 1st month, and 2nd month after the surgery, the patient was pain-free with no postoperative complications. The patient's recovery was uneventful, and he returned to his normal activities within a week after the surgery.

## 3 Discussion

Many physicians question the existence of chronic appendicitis, and it has posed a therapeutic puzzle [7]. The proposed cause of chronic appendicitis is partial or brief obstruction of the appendiceal lumen due to fecalith, tumor, lymphoid hyperplasia, foreign body, or appendiceal mucosal folding. However, the exact pathophysiology is not well elucidated in the literature [3,5,7]. Lack of well-developed diagnostic criteria coupled with milder symptoms of the case usually results in misdiagnosis or delay in diagnosis, prolonging patients suffering [5]. Some authors propose a chronic or recurring abdominal pain lasting more than seven days to distinguish chronic from acute appendicitis [8]. Others suggested a symptom that lasts for more than two weeks with proven features of chronic inflammation on pathologic exam and resolution of the pre-operative symptoms after appendectomy as defining criteria for chronic appendicitis [1,10,12].

Even though there is a lack of well-developed diagnostic criteria, authors recommend considering chronic appendicitis in the differential diagnosis of patients presenting with chronic abdominal pain [1,3,4,7,8,10,12,13]. However, it may not be possible to put chronic appendicitis as an initial diagnosis based on clinical presentation alone. In several cases, the ultimate diagnosis is made based on pathologic exam findings [5,14,15]. Many scholars suggest that no specific laboratory or imaging test ultimately establishes the diagnosis [1,10]. Ultrasound, barium enema, and abdominal CT scan can support the diagnosis of chronic appendicitis [7]. Ultrasound is the initial imaging modality used frequently to assess a patient with suspected appendicitis because of its availability, lack of ionizing radiation, and its high sensitivity (93 %) and moderate specificity (83 %) [4,16]. However, there is no agreement regarding the specific ultrasound feature of chronic appendicitis [16].

Nevertheless, in our case, abdominal U/S was done by two different radiologists, and the appendix was only partially compressible has diffuse appendiceal wall thickening with prominent and hyperechogenic submucosa, a finding consistent with appendicitis in both cases. An abdominal CT scan is a modality of choice for chronic appendicitis. It has a feature characterized by a dilated and thickened appendix with inflammatory changes around the pericecal region, similar to acute appendicitis [16]. The possible differentials in a patient presenting with chronic right lower quadrant abdominal pain includes; inflammatory bowel diseases, tuberculosis, lymphoma, ureteric colic, diverticulitis of ascending colon and gynecologic problems in female patients [1]. Abdominal CT scan is important imaging modality in describing these possible differentials and it is most accurate test for equivocal cases [3,8,16].

The pathologic exam finding in chronic appendicitis is characterized by the presence of chronic inflammation with infiltration of lymphocyte, eosinophil, and plasma cells, granulomatous reaction, and fibrosis of the wall of the appendix, which is different from acute appendicitis where mucosal hyperemia and polymorphonuclear cell infiltration is usually noted [1,5,7]. On the other hand, recurrent appendicitis presents with chronic or recurrent abdominal pain but will have histopathologic findings similar to acute appendicitis [1,12]. In our case, the patient presented with chronic right lower quadrant abdominal pain, and the histopathology exam showed the presence of mononuclear inflammatory infiltrates, including lymphocytes and plasma cells with associated trans-mural fibrosis. This finding aligns with chronic appendicitis.

Chronic appendicitis is not considered a surgical emergency, contrary to acute appendicitis [3]. However, earlier diagnosis can prevent the development of related complications and patients suffering from the pain [13,15]. Elective appendectomy should be done for such patients, and the vast majority of them will have a smooth recovery with the resolution of their symptoms [3,8]. Our patient presented with a complaint of chronic abdominal pain, and the presumptive diagnosis of chronic appendicitis was made after clinical and abdominal U/S evaluation. An appendectomy was done, and the diagnosis of chronic appendicitis was confirmed with histopathologic examination. The patient was seen in the follow-up clinic and is relieved from his symptoms.

## 4 Conclusion

Chronic appendicitis can have an atypical presentation with milder symptoms and should be considered as a differential in patients suffering from chronic right lower quadrant abdominal pain with associated tenderness. Early diagnosis and prompt intervention can decrease the risk of development of complications and prevent patient suffering. Abdominal U/S is an important diagnostic tool in assessing suspected cases of chronic appendicitis, and appendectomy is an effective treatment option to relieve chronic pain.

## Consent

Written informed consent was obtained from the patient to publish this case report and accompanying images. A copy of the written consent form is available for review by the editor-in-chief of this journal upon request.

## Ethical approval

Ethical clearance was not necessary as the format of this paper is case report. The authors' institution IRB has exempted case reports from requiring ethical clearance.

## Guarantor

Dr. Eskinder Amare Assefa

## Research registration number

Not needed.

## Funding

No funding was received for this case report.

## Author contribution

Dr. Eskinder Amare Assefa (MD, Assistant Professor of General Surgery): diagnosed the case and operated the patient, Manuscript writing, and Submission and followed the patient.

Dr. Yonas Girma Shumiye (MD, Assistant Professor of Pathology): Assisted histologic diagnosis of the case and prepared the histologic image of the case.

Dr. Abel Shiferaw Tesfaye (MD, Assistant Professor of General and Colorectal Surgery): Involved in conceptualization of the case and reviewed the final manuscript.

Dr. Anatia Kifle Alemu (MD, MSc in clinical trial): Reviewed the final manuscript and edited the case report.

Dr. Zekarias Seifu Ayalew(MD, Internist):Assisted in the manuscript writing and edited the case report.

## Conflict of interest statement

We don't have any conflict of interest.
